# Diagnosis of Breast Cancer Using Radiomics Models Built Based on Dynamic Contrast Enhanced MRI Combined With Mammography

**DOI:** 10.3389/fonc.2021.774248

**Published:** 2021-11-17

**Authors:** You-Fan Zhao, Zhongwei Chen, Yang Zhang, Jiejie Zhou, Jeon-Hor Chen, Kyoung Eun Lee, Freddie J. Combs, Ritesh Parajuli, Rita S. Mehta, Meihao Wang, Min-Ying Su

**Affiliations:** ^1^ Department of Radiology, First Affiliated Hospital of Wenzhou Medical University, Wenzhou, China; ^2^ Department of Radiological Sciences, University of California, Irvine, Irvine, CA, United States; ^3^ Department of Radiology, E-Da Hospital and I-Shou University, Kaohsiung, Taiwan; ^4^ Department of Radiology, Inje University Seoul Paik Hospital, Inje University, Seoul, South Korea; ^5^ Department of Medicine, University of California, Irvine, Irvine, CA, United States; ^6^ Department of Medical Imaging and Radiological Sciences, Kaohsiung Medical University, Kaohsiung, Taiwan

**Keywords:** breast neoplasms, diagnosis, radiomics, machine learning, magnetic resonance imaging, mammography

## Abstract

**Objective:**

To build radiomics models using features extracted from DCE-MRI and mammography for diagnosis of breast cancer.

**Materials and Methods:**

266 patients receiving MRI and mammography, who had well-enhanced lesions on MRI and histologically confirmed diagnosis were analyzed. Training dataset had 146 malignant and 56 benign, and testing dataset had 48 malignant and 18 benign lesions. Fuzzy-C-means clustering algorithm was used to segment the enhanced lesion on subtraction MRI maps. Two radiologists manually outlined the corresponding lesion on mammography by consensus, with the guidance of MRI maximum intensity projection. Features were extracted using PyRadiomics from three DCE-MRI parametric maps, and from the lesion and a 2-cm bandshell margin on mammography. The support vector machine (SVM) was applied for feature selection and model building, using 5 datasets: DCE-MRI, mammography lesion-ROI, mammography margin-ROI, mammography lesion+margin, and all combined.

**Results:**

In the training dataset evaluated using 10-fold cross-validation, the diagnostic accuracy of the individual model was 83.2% for DCE-MRI, 75.7% for mammography lesion, 64.4% for mammography margin, and 77.2% for lesion+margin. When all features were combined, the accuracy was improved to 89.6%. By adding mammography features to MRI, the specificity was significantly improved from 69.6% (39/56) to 82.1% (46/56), p<0.01. When the developed models were applied to the independent testing dataset, the accuracy was 78.8% for DCE-MRI and 83.3% for combined MRI+Mammography.

**Conclusion:**

The radiomics model built from the combined MRI and mammography has the potential to provide a machine learning-based diagnostic tool and decrease the false positive diagnosis of contrast-enhanced benign lesions on MRI.

## Introduction

Breast cancer is the most common cancer in women, and one main cause of cancer deaths (1, 2). Mammography, ultrasound, and magnetic resonance imaging (MRI) are well-established diagnostic modalities, which are known to reveal different aspects of underlying abnormalities and provide complementary information for diagnosis (3, 4). Dynamic contrast-enhanced MRI (DCE-MRI) can assess angiogenesis (5, 6), which is essential for cancer development and progression (7, 8). The high spatial resolution and 3D imaging capability of MRI allow for detecting early small cancers, and for evaluating the extent of the disease for pre-operative staging and treatment planning. However, some benign diseases may show strong contrast enhancements and lead to a false positive diagnosis (9).

Mammography can detect breast cancer based on the presence of mass, microcalcifications, architectural distortion, or asymmetric density. It is a widely used imaging modality for screening and diagnosis, and crucial for detecting breast cancer at an early, curable, stage to decrease mortality (10). However, mammography is limited by breast density, which may compromise the detection sensitivity. For women with a high-risk of developing breast cancer, the screening is recommended to start from a young age, and to mitigate the problem of high density in mammography MRI is commonly used as a supplementary modality. Since different imaging can evaluate different pathological characteristics of the abnormal tissue, combining them may improve the diagnostic accuracy (3). MRI is also commonly used for problem-solving when other imaging shows equivocal findings. For example, in patients with category 4 mammographic microcalcifications, MRI can decrease false positive findings and unnecessary biopsy (11).

Breast Imaging Reporting and Data System (BI-RADS) (12) is used to indicate the level of suspicion in detected abnormality. However, subjective reading using the BI-RADS lexicon only achieved moderate levels of inter-reader agreement (13). For MRI, intra-/inter-observer agreement was particularly worse for non-mass enhancement compared to mass lesions (14, 15). To circumvent this problem, computer-aided diagnosis (CAD) systems have been proposed to develop quantitative models that are not subject to high variations to serve as potential diagnostic tools (16, 17).

Artificial intelligence (AI) based radiomics study has been widely applied for medical applications. The method allows for high-throughput extraction of quantitative features from radiographic images (18), and it has been shown as a feasible approach for diagnosis of breast cancer using mammography (19–22) and MRI (23–25). However, the combined model using different imaging modalities was rarely reported. Features from corresponding lesions on each modality can be extracted, and then combined in the selection process to develop better models based on their complementary information.

The purpose of this study was to evaluate the diagnostic performance of radiomics models built based on DCE-MRI and mammography. The motivation was coming from the high false positive diagnosis of contrast-enhanced benign lesions commonly seen on MRI. It is anticipated that the complementary information provided by the radiomics analysis of the lesion on mammography may help to improve the diagnostic accuracy. In mammography, features extracted from the lesion and the margin were used to build separate models. The complementary role of MRI and mammography was first evaluated by the selected features, and then by comparing the performance of final models built using each modality alone and in combination.

## Material and Methods

### Study Population

This retrospective study was approved by Institutional Review Board and written informed consent was waived. Earlier patients who received DCE-MRI and mammography for diagnosis between July 2017 and August 2019 and had confirmed pathology were retrospectively identified as the training set. Later patients from September 2019 to July 2020 were used as the independent testing set. The exclusion criteria were: (1) no pathology result; (2) not visible on MRI or mammography; (3) having prior surgery, chemotherapy, or other treatment; (4) the interval between the two examinations longer than one month; (5) poor image quality. Finally, a total of 268 lesions were included, 202 lesions (146 malignant and 56 benign) in the training set, and 66 lesions (48 malignant and 18 benign) in the testing set. The BI-RADS scores of MRI and mammography were obtained from the radiology reports, classified into 2, 3, 4A, 4B, 4C, and 5. In our institution, BI-RADS 4 MRI cases were routinely subdivided to 4A, 4B, and 4C, as validated in Strigel et al. (26).

### Image Acquisition

Mammography was performed using Fujifilm Amulet Innovality Digital Mammography System with a resolution of 5828×4728 pixels, including craniocaudal (CC) and mediolateral oblique (MLO) view. MRI was performed on a 3.0T scanner (GE SIGNA HDx) using a dedicated 8-channel bilateral breast coil. The imaging protocol included axial and sagittal T2- and T1-weighted sequences, and the DCE acquisition performed using the volume imaging for breast assessment (VIBRANT) sequence. The parameters were: repetition time= 5msec, echo time= 2msec, flip angle= 10°, slice thickness= 1.2mm, field of view= 34×34cm^2^, matrix size= 416×416, temporal resolution= 90sec, and total scan time= 9min. The DCE series consisted of 6 frames: one pre-contrast and 5 post-contrast. The contrast agent, 0.1 mmol/kg body weight of gadopentetate dimeglumine (Magnevist; Bayer Schering Pharma), was injected after the pre-contrast images were acquired, with a flow rate of 2 mL/s followed by a flush of 20 mL saline.

### Tumor Segmentation

For MRI, the tumor region of interest (ROI) segmentation was done using computer algorithms, according to the location and the range of slices. The fuzzy-C-means clustering algorithm was applied to perform segmentation on each DCE slice containing the lesion. The automatic segmentation results were evaluated by two radiologists separately, and adjusted if necessary. Then, the ROIs from all slices were combined, and the 3D connected-component labeling and the hole-filling algorithms were applied to generate the final 3D mask (27, 28). For the corresponding mammography, two radiologists manually outlined the lesion on craniocaudal (CC) or mediolateral oblique (MLO) view by consensus using ITK-SNAP software (version 3.8, www.itksnap.org), with the guidance of the lesion shown on the maximum intensity projection (MIP) of MRI, projected from different angles. The choice of CC or MLO was determined according to the lesion visibility, and only one view was used.

### MRI and Mammography Radiomics Feature Extraction

**Figure 1:**
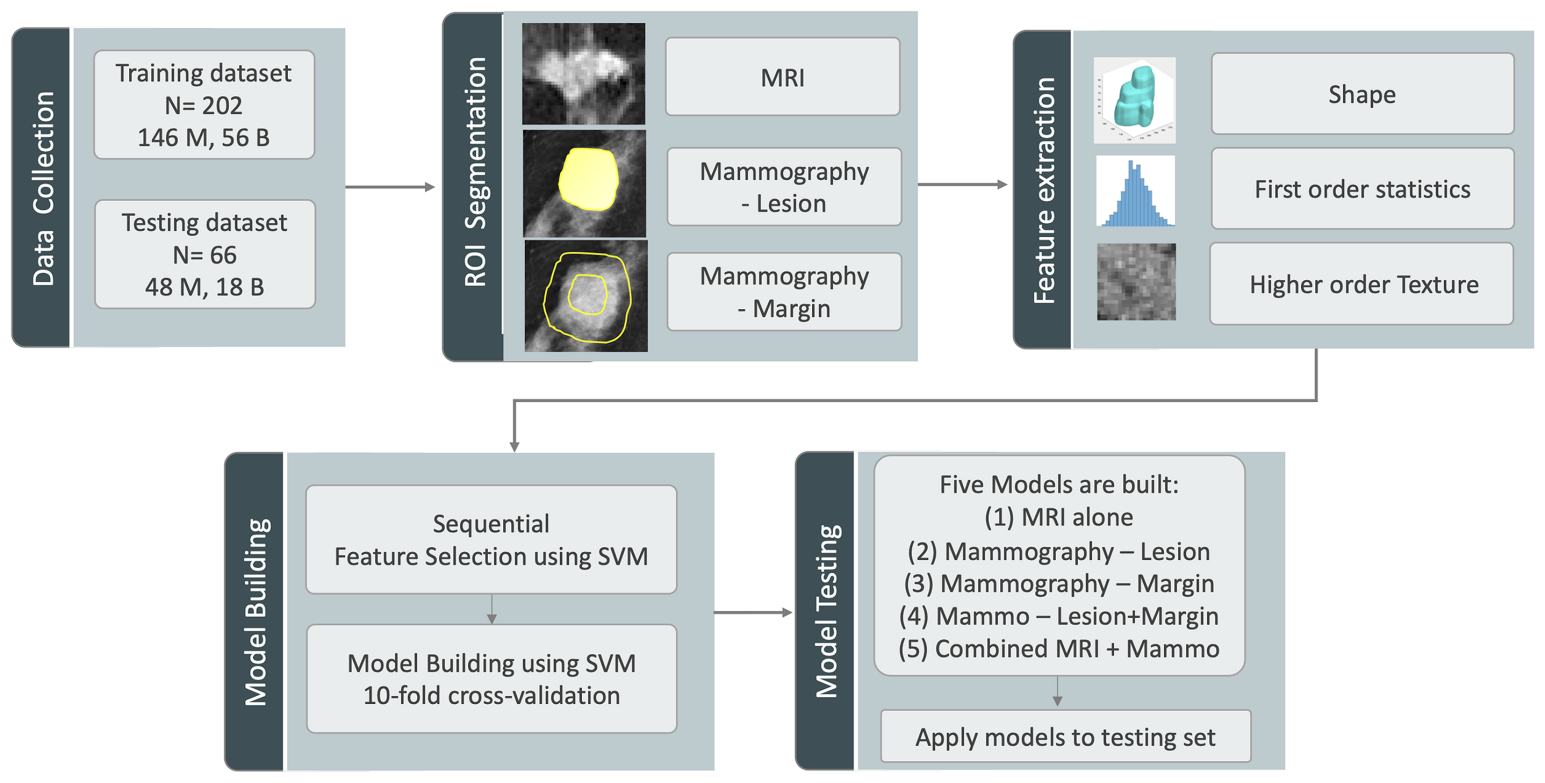
The analysis flowchart. The training and testing sets are assembled according to the time of case enrollment. The analysis starts with ROI segmentation, followed by radiomics feature extraction using Pyradiomics, feature selection and model building in the training set using SVM with cross-validation, and lastly, the testing of the 5 developed models in the testing set.

**Figure 2:**
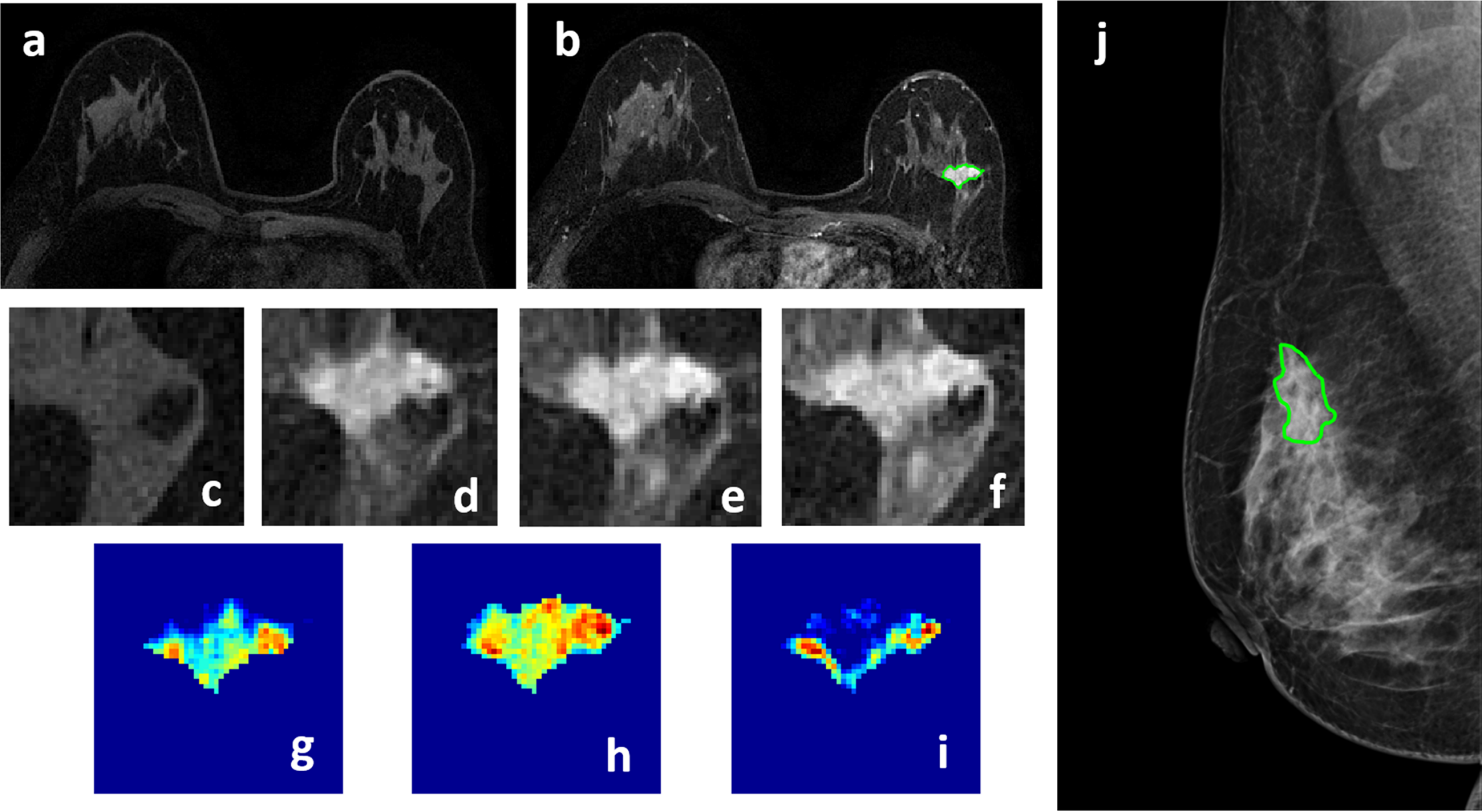
A 50-year-old patient with invasive ductal cancer, showing a strongly enhanced 1.8 x 1.0 cm lesion, with MRI BI-RADS score of 5. **(A)** F1 Pre-contrast image. **(B)** F2 post-contrast image. **(C–I)** Magnified images to demonstrate the margin and internal enhancements within the lesion. **(C)** F1 pre-contrast, **(D)** F2 post-contrast, **(E)** F3 post-contrast, **(F)** The last F6 post-contrast image. **(G)** The wash-in signal enhancement map F2-F1, **(H)** The F3-F1 signal enhancement map, **(I)** The wash-out F6-F3 map. **(J)** A mass lesion with spiculation is clearly noted on mammography as BI-RADS 4C, and manually outlined by a radiologist. The radiomics malignancy probability predicted by MRI, mammography, and combined models were: 0.83, 0.77, 0.88, respectively, true positive.

**Figure 3:**
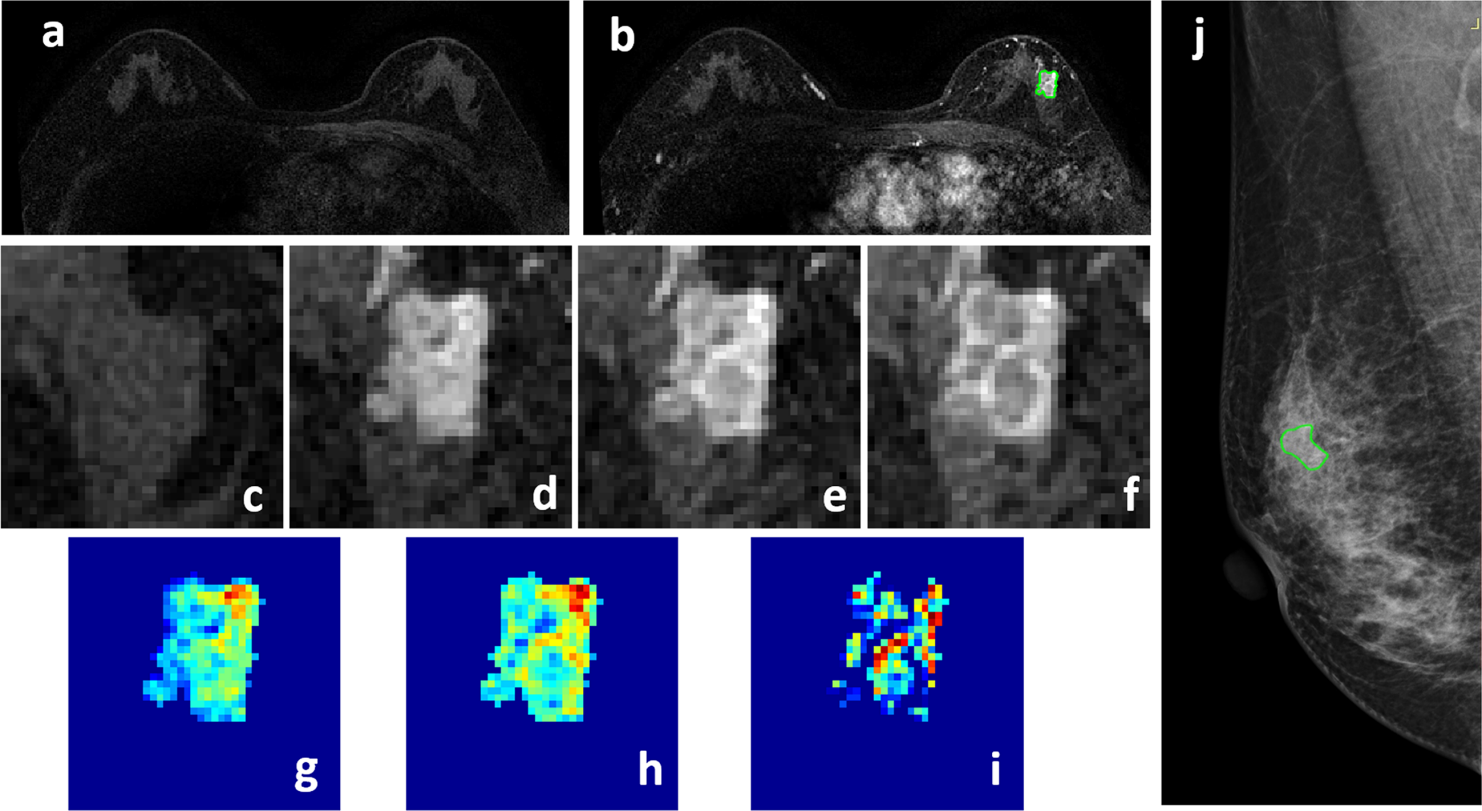
A 58-year-old patient with ductal carcinoma *in situ*, showing a strongly enhanced heterogeneous 1.4 x 0.9 cm lesion, with MRI BI-RADS score of 5. **(A)** F1 Pre-contrast image. **(B)** F2 post-contrast image. **(C–I)** Magnified images to demonstrate the margin and internal enhancements within the lesion. **(C)** F1 pre-contrast, **(D)** F2 post-contrast, **(E)** F3 post-contrast, **(F)** The last F6 post-contrast image. **(G)** The wash-in signal enhancement map F2-F1, **(H)** The F3-F1 signal enhancement map, **(I)** The wash-out F6-F3 map. **(J)** A suspicious BI-RADS 4A mass is seen on mammography. The lesion ROI is outlined with the guidance of MRI. The probability predicted by MRI, mammography, and combined radiomics models were: 0.53, 0.49, 0.62, respectively, true positive.

**Figure 4:**
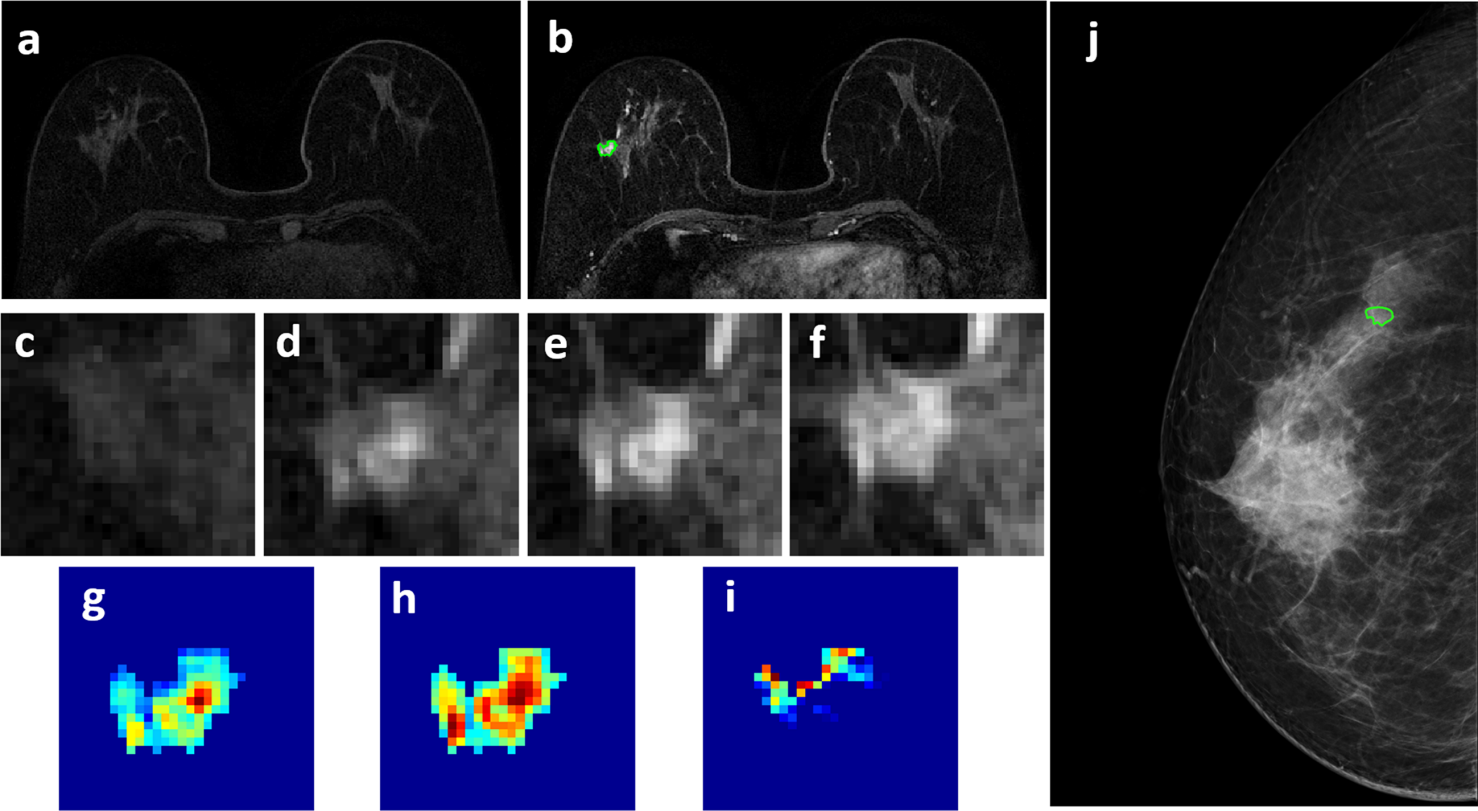
A 63-year-old patient with a 0.7 x 0.7 cm benign adenosis, showing a persistent DCE-MRI enhancement kinetics and determined as BI-RADS 3 on MRI. **(A)** F1 Pre-contrast image. **(B)** F2 post-contrast image. **(C–I)** Magnified images to demonstrate the margin and internal enhancements within the lesion. **(C)** F1 pre-contrast, **(D)** F2 post-contrast, **(E)** F3 post-contrast, **(F)** The last F6 post-contrast image. **(G)** The wash-in signal enhancement map F2-F1, **(H)** The F3-F1 signal enhancement map, **(I)** The wash-out F6-F3 map. **(J)** The lesion is not seen on mammography, determined as BI-RADS 2, and an area is outlined with the guidance of MRI. The probability predicted by MRI, mammography, and combined radiomics models were: 0.42, 0.44, 0.15, respectively, true negative.

**Figure 5:**
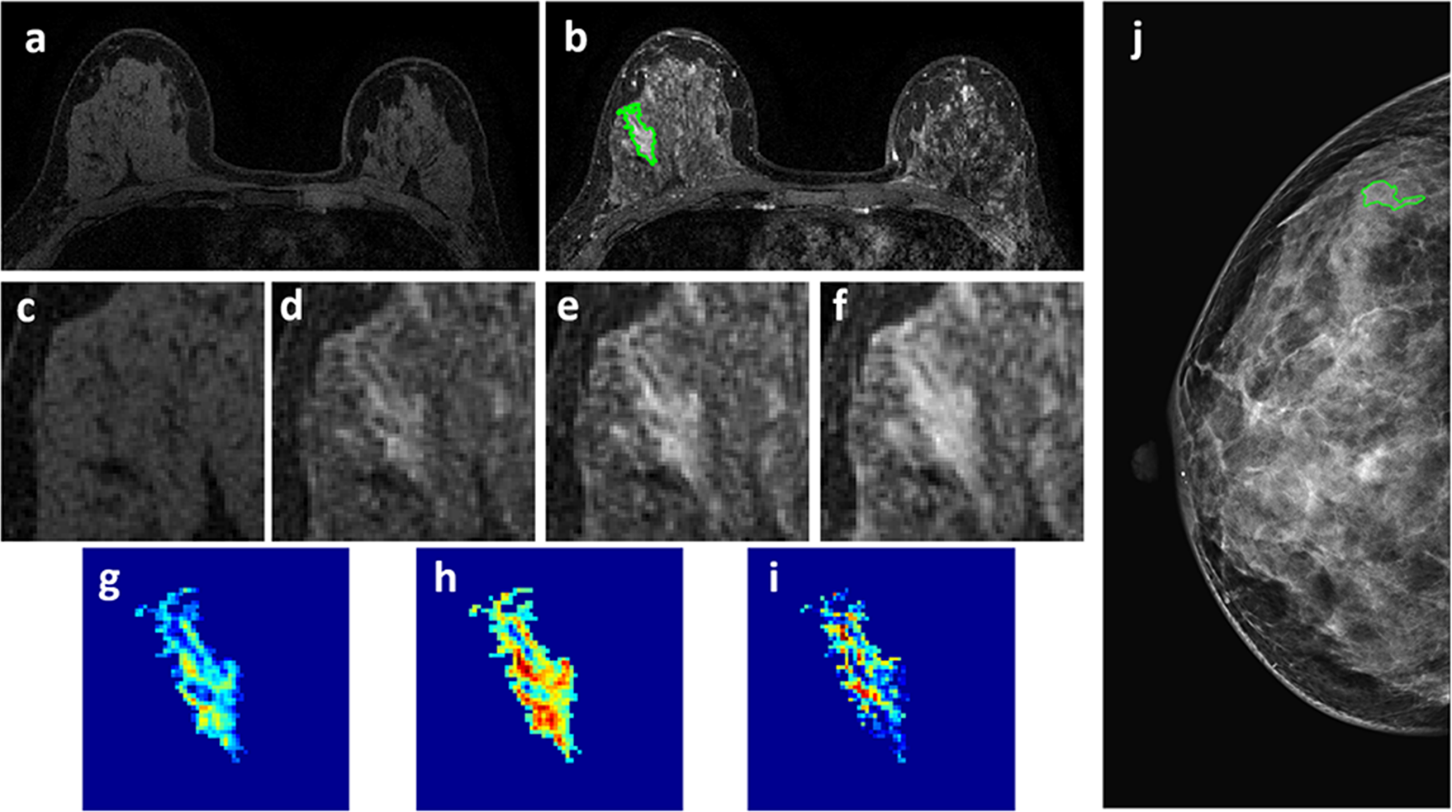
A 46-year-old patient with a 2.7 x 1.3 cm benign adenosis. This is a young woman with extremely dense breasts showing substantial parenchymal enhancements. The lesion shows a persistent DCE-MRI pattern and determined as BI-RADS 4A on MRI. **(A)** F1 Pre-contrast image. **(B)** F2 post-contrast image. **(C–I)** Magnified images to demonstrate the margin and internal enhancements within the lesion. **(C)** F1 pre-contrast, **(D)** F2 post-contrast, **(E)** F3 post-contrast, **(F)** The last F6 post-contrast image. **(G)** The wash-in signal enhancement map F2-F1, **(H)** The F3-F1 signal enhancement map, **(I)** The wash-out F6-F3 map. **(J)** The lesion is not seen on mammography, determined as BI-RADS 2, and an area is outlined with the guidance of MRI. The probability predicted by MRI, mammography, and combined radiomics models were: 0.3, 0.41, 0.11, respectively, true negative.

The analysis flowchart is demonstrated in **Figure 1**. For DCE-MRI, three heuristic DCE parametric maps were generated according to: the early wash-in signal enhancement (SE) ratio ((F2-F1)/F1); the maximum SE ratio = ((F3-F1)/F1); the wash-out slope ((F6-F3)/F3) (25), as illustrated in case examples in **Figures 2**–**5**. The intensity was normalized to mean=0 and standard deviation=1. In the segmented 3D ROI, pixels were transformed into isotropic 0.82×0.82×0.82 mm by B-spline interpolation. The radiomics analysis was performed using the PyRadiomics, an open-source radiomics library written in Python (29). On each parametric map, a total of 107 features were extracted, including 14 shape, 18 first-order, 24 gray-level co-occurrence matrix (GLCM), 14 gray-level dependence matrix (GLDM), 16 gray-level run length matrix (GLRLM), 16 gray-level size zone matrix (GLSZM), and 5 neighboring gray tone difference matrix (NGTDM) features, so there was a total of 321 parameters from 3 maps. Only 268 features showing intra-class coefficient (ICC) ≥0.8 were included in the final analysis, which was determined using two sets of separately segmented tumor ROI to evaluate the reproducibility of extracted radiomics features (30).

For mammography, two different feature sets were analyzed. Considering that the ROI was manually drawn by tracing the visible lesion area based on density, it might not reveal the margin information. To specifically focus on the margin, a 2-cm bandshell was created, by shrinking and expanding the manually-drawn tumor boundary by 1 cm, as shown in **Figure 1**. Because the margin could not be well defined on mammography, shrinking the boundary followed by region growing has been shown as a feasible segmentation method (31), and the method was adopted here to generate the bandshell for analysis of margin features. Similarly, the intensity was normalized to mean=0 and standard deviation=1, and a total of 107 PyRadiomics features were extracted from the outlined lesion mask and also from the bandshell on mammography. The radiomics model was first performed using lesion features alone, margin features alone, and then a combined model was built by considering all lesion and margin features.

### Feature Selection and Model Building in Training Set

The procedures are also shown in **Figure 1**. In addition to the normalization on images, each feature extracted from all cases was normalized to mean=0 and standard deviation=1 before training. To evaluate the importance of these features in diagnosis, a sequential forward feature selection method using the support vector machine (SVM) was applied (32, 33). In this process, we used SVM with Gaussian kernel as the objective function to test the performance of models built with a subset of features. In the beginning, an empty candidate set was presented, and features were sequentially added. The 10-fold cross-validation was applied to test the model performance. In each iteration, the training process was repeated 1,000 times to explore the robustness of each feature. After each iteration, the feature which led to the best performance was added to the candidate set. When the addition of features no longer met the criterion, the selection process stopped. Here, we used 10*e^-6 as termination tolerance for the objective function value.

The selected features were used to build the SVM classification model with Gaussian kernel to classify the benign and malignant groups. The diagnostic performance was tested using 10-fold cross-validation. Each case had only one chance to be included in the validation set. The probability of all cases in the validation set was combined to perform the receiver operating characteristic curve (ROC) analysis, and the area under the curve (AUC) was calculated. Five models were built using features extracted from: 1) DCE-MRI; 2) mammography – lesion ROI; 3) mammography – margin ROI, i.e., the bandshell; 4) mammography lesion+margin; and 5) all combined. The developed model gave a radiomics score, i.e., the malignancy probability, for each case.

### Applying the Trained Models to the Testing Set

The developed models from the training set were applied to test their performances in the testing set. The model gave each lesion a radiomics score, and they were used to generate the ROC curves. The sensitivity, specificity, positive predicting value (PPV), negative predicting value (NPV), and overall accuracy of each model were calculated using the threshold of probability ≥0.5 as malignant. The Delong test was used to compare the difference between paired ROC curves. The difference in proportions between malignant and benign groups was compared by using the Chi-square (χ^2^) test or Fisher’s Exact Test.

## Results

### Patients’ Characteristics and BI-RADS Scores

In the training set, the mean age was 50.0 ± 9.6 in the malignant, and 46.6 ± 9.7 in the benign groups. The 1-D longest dimension tumor size measured on MRI was 2.4 ± 1.4 cm (median 2.0 cm) in the malignant, and 2.0 ± 2.3 cm (median 1.5 cm) in the benign groups. In the testing set, the mean age was 51.8 ± 11.2 in the malignant, and 43.5 ± 10.8 in the benign groups. The 1-D longest dimension tumor size measured on MRI was 3.2 ± 1.9 cm (median 2.8 cm) in the malignant, and 2.0 ± 1.4 cm (median 1.5 cm) in the benign groups. The pathological types and BI-RADS distributions in both datasets are listed in **Table 1**. In the training set, the majority of malignant lesions had BI-RADS scores of 4B, 4C, 5 on MRI (132/146 = 90.4%) and mammography (120/146 = 82.2%). In the benign group, a substantial number of patients also had high BI-RADS ≥ 4B diagnosed by MRI (20/56 = 35.7%) and mammography (16/56 = 28.6%). Although the number of patients with BI-RADS ≥ 4B lesions was significantly smaller in the benign compared to the malignant groups (p < 0.001), these cases would be recommended for biopsy and led to false positive diagnosis. Similar BI-RADS distributions were also noted in the testing set.

**Table 1:** Pathological types and BI-RADS scores of lesions in training and testing datasets.

Characteristics	Training (N = 202)	Testing (N = 66)
Benign	56	18
Fibroadenoma	13 (23.2%)	5 (27.8%)
Adenosis	25 (44.6%)	10 (55.6%)
Intraductal papilloma	10 (17.9%)	1 (5.6%)
Inflammation	2 (3.6%)	0 (0.0%)
Others	6 (10.7%)	2 (11.1%)
MRI BI-RADS		
2	9 (16.1%)	1 (5.6%)
3	13 (23.2%)	3 (16.7)
4A	14 (25%)	9 (50%)
4B	14 (25%)	4 (22.2%)
4C	5 (8.9%)	1 (5.6%)
5	1 (1.8%)	0 (0.0%)
Mammography BI-RADS		
2	13 (23.2%)	5 (27.8)
3	16 (28.6%)	6 (33.3%)
4A	11 (19.6%)	4 (22.2%)
4B	12 (21.4%)	3 (16.7%)
4C	4 (7.1%)	0 (0.0%)
5	0 (0%)	0 (0.0%)
Malignant	146	48
Invasive ductal cancer	113 (77.4%)	39 (81.3%)
Ductal carcinoma *in-situ*	23 (15.8%)	3 (6.3%)
Intraductal papillary carcinoma	4 (2.7%)	0 (0.0%)
Mucinous carcinoma	3 (2.1%)	1 (2.1%)
Others	3 (2.1%)	5 (10.4%)
MRI BI-RADS		
3	1 (0.7%)	0 (0.0%)
4A	13 (8.9%)	1 (2.1%)
4B	17 (11.6%)	4 (8.3%)
4C	39 (26.7%)	18 (37.5%)
5	76 (52.1%)	25 (52.1%)
Mammography BI-RADS		
2	0	4 (8.3%)
3	9 (6.2%)	1 (2.1%)
4A	17 (11.6%)	2 (4.2%)
4B	32 (21.9%)	11 (22.9%)
4C	48 (32.9%)	21 (43.8%)
5	40 (27.4%)	9 (18.8%)

BI-RADS, Breast Imaging Report and Data System.

### Radiomics Diagnostic Models in Training Set

**Figure 6:**
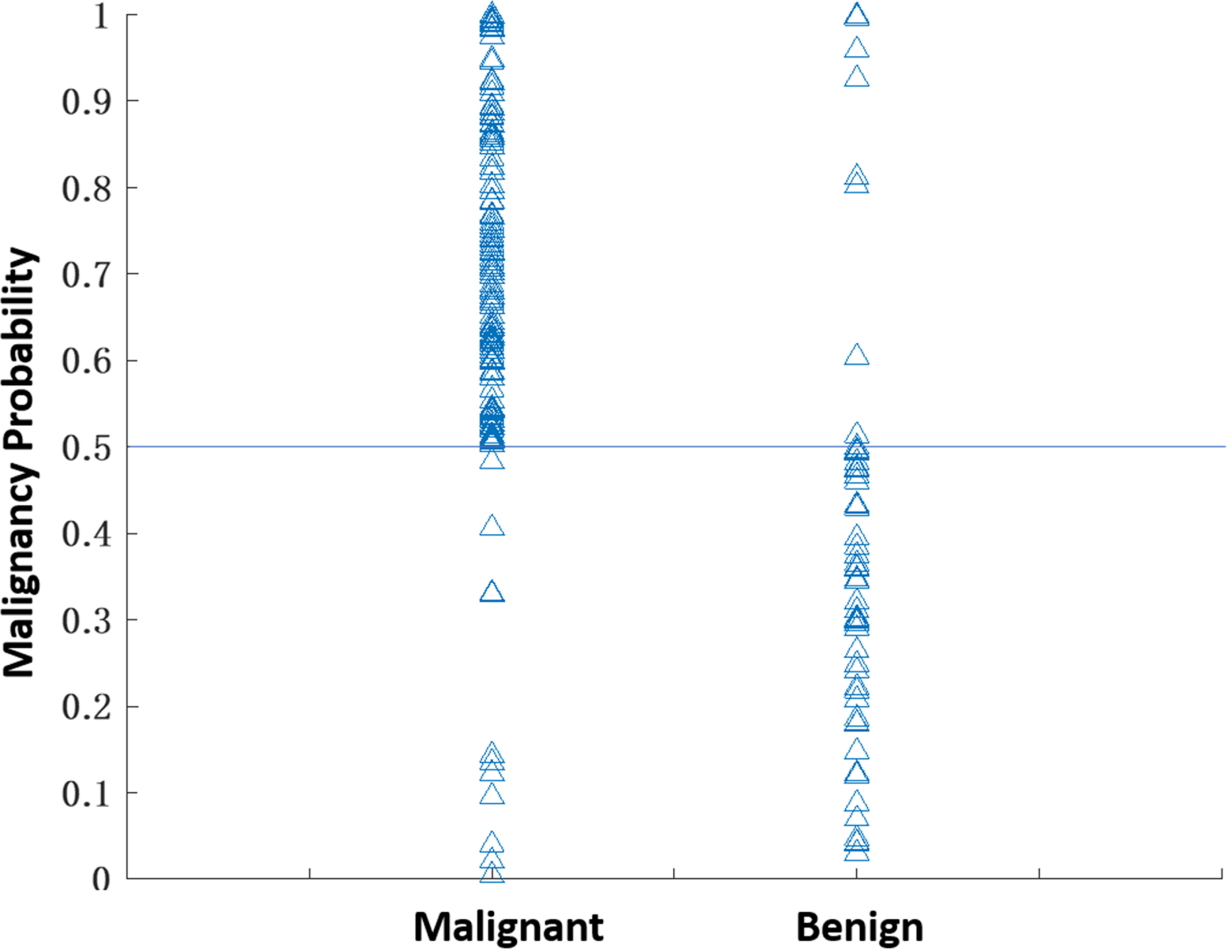
The malignant probability predicted by the combined MRI+Mammography radiomics model in 202 lesions, 146 malignant and 56 benign, in the training set. Using the threshold of 0.5 as the cut-off, there are 135 true positive, 11 false negative, 46 true negative, and 10 false positive cases, with an overall accuracy of 181/202 = 89.6%.

The selected radiomics features for each model are listed in **Table 2**. The diagnostic sensitivity, specificity, PPV, NPV, accuracy, and AUC obtained from the cross-validation results are summarized in **Table 3**. The overall accuracy was 83.2% for DCE-MRI. In mammography, the accuracy was 75.7% for lesion-ROI, 64.4% for margin-ROI, and when combining both of them it was improved to 77.2%. When all MRI and mammography features were combined to build a model, the accuracy was improved to 89.6%, which was significantly better than the mammography model (77.2%, p=0.001). The combined model was also better than the MRI model (83.2%, p=0.059), but not reaching significance. By adding mammography features to MRI, the specificity was significantly improved from 69.6% (39/56) to 82.1% (46/56) (p<0.01), while sensitivity was also improved from 88.4% (129/146) to 92.5% (135/146). **Figure 6** plots the malignant probability predicted by the combined MRI+ Mammography radiomics model in the training set of 146 malignant and 56 benign lesions. Using the threshold of 0.5 as the cut-off, there are 135 true positive, 46 true negative, 11 false negative, and 10 false positive cases, with an overall accuracy of 181/202 = 89.6%.

**Table 2:** Selected radiomics features for modeling using MRI, mammography, and both combined.

Models	Selected Radiomic Features	Numbers
DCE-MRI	**Maximum signal enhancement ratio:**	8
	entropy, GLCM sum average, GLCM IMC1,	
	GLDM high gray level emphasis, skewness	
	**Wash-in ratio:**	
	GLRLM RLN	
	**Wash-out ratio:**	
	GLRLM small area emphasis, GLCM sum entropy	
Mammography	90% value, entropy, GLCM maximum probability, GLDM high gray level emphasis	4
(Lesion)		
Mammography	10% value, GLSZM zone entropy, GLCM IDN	3
(Margin)		
Combination of DCE-MRI and mammography	**Maximum signal enhancement ratio:**	9
	kurtosis, GLCM IMC1	
	**Wash-in ratio:**	
	skewness, GLRLM RLN, NGTDM complexity	
	**Wash-out ratio:**	
	GLCM IMC1, GLCM sum entropy	
	**Mammography lesion:**	
	GLCM maximum probability, GLCM IDN	

GLCM, gray level co-occurrence matrix; GLDM, gray level dependence matrix; GLRLM, gray level run length matrix; GLSZM, gray level size zone matrix; NGTDM, neighboring gray tone difference matrix; IMC, informational measure of correlation; IDN, inverse difference normalized; RLN, run length non-uniformity.

**Table 3:** The diagnostic performance of developed radiomics models in training and testing datasets.

Models	Sensitivity	Specificity	PPV	NPV	Accuracy	AUC
**Training Dataset**						
DCE-MRI	88.4%	69.6%	88.4%	69.6%	83.2%	0.77
	(129/146)	(39/56)	(129/146)	(39/56)		
Mammography	84.9%	51.8%	82.1%	56.9%	75.7%	0.69
(Lesion)	(124/146)	(29/56)	(124/151)	(29/51)		
Mammography	73.3%	41.1%	76.4%	37.1%	64.4%	0.62
(Margin)	(107/146)	(23/56)	(107/140)	(23/62)		
Mammography	84.9%	57.1%	83.8%	59.3%	77.2%	0.70
(Lesion+Margin)	(124/146)	(32/56)	(124/148)	(32/54)		
All Combination	92.5%	82.1%	93.1%	80.7%	89.6%	0.83
	(135/146)	(46/56)	(135/145)	(46/57)		
**Testing Dataset**						
DCE-MRI	87.5%	55.6%	84%	62.5%	78.8%	0.80
	(42/48)	(10/18)	(42/50)	(10/16)		
Mammography	81.3%	38.9%	78%	43.8%	69.7%	0.65
(Lesion)	(39/48)	(7/18)	(39/50)	(7/16)		
Mammography	66.7%	33.3%	59.3%	27.3%	57.6%	0.53
(Margin)	(32/48)	(6/18)	(32/54)	(6/22)		
Mammography	81.3%	38.9%	78%	43.8%	69.7%	0.64
(Lesion+Margin)	(39/48)	(7/18)	(39/50)	(7/16)		
All Combination	91.7%	61.1%	86.3%	73.3%	83.3%	0.81
	(44/48)	(11/18)	(44/51)	(11/15)		

PPV, positive predicting value; NPV, negative predicting value; AUC, the area under the curve; DCE, dynamic contrast enhanced.

Four case examples are shown. **Figure 2** is an IDC with BI-RADS 5 MRI and BI-RADS 4C mammography, and the malignancy probability predicted by MRI, mammography, and combined models are: 0.83, 0.77, 0.88, respectively; thus, true positive. **Figure 3** is a DCIS, also with BI-RADS 5 MRI and a lower BI-RADS 4A mammography, and the combined radiomics probability is 0.62, true positive. **Figure 4** is a very small 0.7 cm benign adenosis with BI-RADS 3 MRI and BI-RADS 2 mammography, and the combined radiomics probability is 0.15, true negative. **Figure 5** is another adenosis in a younger woman with BI-RADS 4A MRI and BI-RADS 2 mammography, and the combined radiomics probability is 0.11, true negative. These cases demonstrate that the malignancy probability predicted by radiomics models was consistent with BI-RADS reading, and elaborate how the model may help to improve the diagnostic confidence.

### Performance of the Trained Models in Testing Set

The developed models were then applied to cases in the independent testing set to test the performance. The results are listed in **Table 3**. In general, the performance of these 5 models was consistent with the validation results in the training set. The accuracy was 78.8% for DCE-MRI, 69.7% for mammography, and improved to 83.3% when using the combined MRI and mammography model.

### Performance of the Combined Model in Each BI-RADS Category

In order to further evaluate the performance of the model in each BI-RADS category, the results from the training and testing sets are combined and listed in **Table 4**. The cases with BI-RADS score of 2, 3, 4A, 4B, 4C, and 5 based on MRI and mammography were separately tabulated. It can be seen clearly that malignant lesions have higher BI-RADS scores compared to benign lesions, but many benign lesions also have ≥4B scores. First, in the malignant group, if we used 2, 3, and 4A as more likely benign, 15 MRI and 33 mammography cases would be diagnosed as benign. The results showed that the model could reach 14/15 = 93.3% accuracy for MRI and 31/33 = 93.9% for mammography lesions, still with a high sensitivity. On the other hand, in the benign group, if we used 4B, 4C and 5 as possibly malignant, 25 MRI and 19 mammography cases would be diagnosed as malignant. The model could achieve 18/25 = 72% accuracy for MRI and 15/19 = 78.9% for mammography lesions. The correct benign diagnosis for these cases may help to avoid unnecessary biopsy.

**Table 4:** The number of correctly diagnosed cases made by the combined radiomics model in each BI-RADS category.

BI-RADS Score	Malignant Cases (N = 194)		Benign Cases (N = 74)	
	MRI	Mammography	MRI	Mammography
2	0	3/4	8/10	15/18
3	1/1	9/10	11/16	18/22
4A	13/14	19/19	20/23	9/15
4B	19/21	38/43	13/18	11/15
4C	51/57	67/69	4/6	4/4
5	95/101	43/49	1/1	0

BI-RADS, Breast Imaging Report and Data System.

## Discussion

In this study, we developed the radiomics models for diagnosis of breast cancer using DCE-MRI alone, mammography alone, and the combined MRI and mammography. While quite a few studies have reported the radiomics models developed using MRI (23, 24, 34) or mammography (19–22), the combined analysis was rarely reported (35). We further investigated the complementary role of MRI and mammography features in diagnostic sensitivity and specificity. In the training set, the combined model (89.6%) had a higher accuracy than individual ones (83.2% for mammography, 77.2% for mammography). When mammography features were added to MRI features, it could significantly improve specificity from 69.6% (39/56) to 82.1% (46/56); and thus, have the potential to decrease unnecessary biopsy. Interestingly, the sensitivity was also improved, so the higher specificity was not at the expense of compromised sensitivity. Similar findings were seen in the testing set, with slightly lower overall accuracy from 89.6% to 83.3%.

For mammography, we further separated the analysis using features extracted from the lesion-ROI alone, and from the margin-ROI alone by using a bandshell. The results showed that the accuracy was much better for the lesion model than the margin model, but the margin information could help to improve the accuracy. The results were consistent with the knowledge that margin plays an important role in characterization of a lesion for diagnosis.

Since MRI is more expensive than mammography, the most established clinical indication is for pre-operative staging and high-risk screening. It is not always included in the standard diagnostic workup. It has been shown that in the mammography 4 category, particularly in non-palpable lesions presenting only with microcalcifications, MRI can be used to reduce false positives and avoid unnecessary biopsy (11, 36, 37). On the other hand, benign lesions may show enhancements on MRI, and the information from mammography may help to rule out malignancy (38). As in the case examples shown in **Figures 4** and **5**, the benign lesions might be inconspicuous on mammography and had low BI-RADS score of 2, and we had to use MIP generated from MRI as a reference to locate them. Since MRI and mammography evaluate different aspects of the underlying pathology, they should be reviewed together to determine which information needs to be weighted more.

Radiomics is becoming an active research field in breast cancer diagnosis. Due to the large number of images acquired using different MR sequences, radiomics provides an efficient analysis method to extract information. Therefore, more MRI radiomics studies were reported than ultrasound, mammography, and 18F FDG PET/CT (34). MRI radiomics was shown to provide better discrimination than conventional parameters for the diagnosis of breast cancer (23, 24). Mammography radiomics analysis has also been performed in several diagnostic studies (19–22). However, since the patient cohort is different, the diagnostic accuracy will be highly dependent on the inclusion/exclusion criteria, and not directly comparable among studies. Mao et al. (19) used four modeling algorithms, including SVM, naive Bayes classifier, k-NN classifier, and logistic regression to differentiate between benign and malignant cases, and showed a high vibration of 0.629-0.978 in the obtained accuracy. The radiologists’ reading accuracy was 0.772. Lei et al. (20) applied radiomics to diagnose patients showing BI-RADS 4 calcifications on mammography, and achieved AUC of 0.80 in the validation cohort. For characterizing microcalcifications, since the lesion area was not well-defined, the ROI drawing will affect the extracted features, and thus, the diagnostic results. Huang et al. (21) applied mammography radiomics for distinguishing male malignant and benign lesions, and reported an AUC of 0.82 – a very unique study in rarely reported male patients. Another study by Niu et al. (22) also analyzed patients showing abnormal lesions on mammography and MRI, close to our patient cohort, but their goal was to evaluate the combined effect of mammography and digital breast tomosynthesis (DBT), as well as the combined effect of DCE and diffusion weighted MRI. The reported accuracy based on the mammography was close to ours, around 0.70. Multi-modal radiomics combining different imaging modalities are rarely reported. In a study by Chen et al., the multimodal classifier achieved a better diagnostic performance than any single modality (35). Since each imaging modality is unique in its acquisition method and parameter setting, the extracted features from a lesion may be different and provide complementary information to improve diagnostic accuracy.

In this study, the cases were identified from the MRI database first, and then only those with mammography performed within one month were further selected for analysis. All lesions showed strong enhancements on MRI, and the information was used to determine a corresponding ROI on mammography. Co-registration of MRI and mammography to ensure that the traced ROI is indeed coming from the same suspicious tissue is not a trivial task. We used maximum intensity projection of MRI as guidance, and it could be projected from different angles to simulate CC view and MLO view to guide the tracing of the suspicious tissues on mammography. Some computer techniques have been proposed for registration between MRI and mammography, e.g., using finite element methods by Hopp et al. (39) and Mertzanidou et al. (40), and the thin-plate spline method by Yang et al. (3). These registration techniques can be considered in future multi-modality radiomics studies. However, since the mammography was acquired using heavily compressed breast tissues in a different body position, it might be difficult to find the precise correspondence. Therefore, in this study we only analyzed the CC or MLO view that had more clear presentation of the lesion.

There were several limitations in this study. First, the models were developed using a dataset from a single institution. The earlier cases were used for training, and the performance was evaluated using 10-fold cross-validation. We assembled an independent testing set using later cases according to time of enrollment, so the developed models from training can be independently tested. Another limitation is that the sample size was relatively small. In our dataset, all benign lesions had to show visible enhancements on MRI and were histologically confirmed, which were very strict criteria and limited the number of eligible cases. However, since the major goal of this study is to investigate whether and how much the addition of mammography radiomics features can complement MRI, using a strict rule to identify eligible cases with histologically confirmed lesions is needed. Third, while all lesions showed enhancements on MRI, lesions not visible with the MRI-guidance on mammography were not included in this study. Since the boundary of these lesions could not be clearly defined, the radiomics features might not be reliably extracted.

In conclusion, the radiomics models built based on combined MRI and mammography had better diagnostic accuracy than models built using single modality alone. The combined model could reach the accuracy of 89.6% in the training and 83.3% in the testing sets. The motivation of this study is to use the complementary information extracted from radiomics analysis of the lesion shown on mammogram to decrease the false positive diagnosis of contrast-enhanced benign lesions on MRI. In the western countries, breast MRI is recommended as a clinical modality for screening of women with a high risk of developing breast cancer, and the false positive diagnosis in a screening population will lead to many unnecessary procedures including biopsy, and patient anxiety. Our study may provide a helpful computer-aided diagnostic tool for such clinical indications. The multimodality radiomics analysis by combining mammography and MRI features has the potential to improve the specificity and reduce unnecessary biopsies, while maintaining a high sensitivity for diagnosis of breast cancer.

## Data Availability Statement

The datasets used and analyzed in this study will be made available by the corresponding author on a reasonable request.

## Ethics Statement

The studies involving human participants were reviewed and approved by Ethics Committee of The First Affiliated Hospital of Wenzhou Medical University. Written informed consent for participation was not required for this study in accordance with the national legislation and the institutional requirements.

## Author Contributions

Study concept and design: Y-FZ and M-YS. Acquisition of data: ZC, JZ, and MW. Analysis of data: YZ, ZC and KL. Drafting of the manuscript: Y-FZ, J-HC, FC, RP, and RM. Critical revision: MW and M-Y S. Statistical analysis: YZ and ZC. Study supervision: MW and M-Y S. All authors contributed to the article and approved the submitted version.

## Funding

This work was supported in part by Foundation of Wenzhou Science & Technology Bureau (No. Y20180185), Medical Health Science and Technology Project of Zhejiang Province Health Commission (No. 2019KY102), Research Incubation Project of First Affiliated Hospital of Wenzhou Medical University (No. FHY2019085), the National Cancer Institute of the National Institutes of Health under award number P30 CA062203, R01 CA127927, R21 CA208938 and the UC Irvine Comprehensive Cancer Center using UCI Anti-Cancer Challenge funds.

## Author Disclaimer

The content is solely the responsibility of the authors and does not necessarily represent the official views of the National Institutes of Health or the Chao Family Comprehensive Cancer Center.

## Conflict of Interest

The authors declare that the research was conducted in the absence of any commercial or financial relationships that could be construed as a potential conflict of interest.

## Publisher’s Note

All claims expressed in this article are solely those of the authors and do not necessarily represent those of their affiliated organizations, or those of the publisher, the editors and the reviewers. Any product that may be evaluated in this article, or claim that may be made by its manufacturer, is not guaranteed or endorsed by the publisher.

## Abbreviations

AI, artificial intelligence; AUC, the area under the curve; BI-RADS, Breast Imaging Report and Data System; CAD, computer-aided diagnosis; CC, cranio-caudal; DCE, dynamic contrast enhanced; DCIS, ductal carcinoma *in-situ*; GLCM, gray-level co-occurrence matrix; GLDM, gray-level dependence matrix; GLRLM, gray-level run length matrix; GLSZM, gray-level size zone matrix; IDC, invasive ductal cancer; MIP, maximum intensity projection; MLO, medio-lateral oblique; MRI, magnetic resonance imaging; NGTDM, neighboring gray tone difference matrix; NPV, negative predicting value; PPV, positive predicting value; ROC, receiver operating characteristic; ROI, region of interest; SE, signal enhancement; SVM, support vector machine.
